# Highly efficient broadband second harmonic generation mediated by mode hybridization and nonlinearity patterning in compact fiber-integrated lithium niobate nano-waveguides

**DOI:** 10.1038/s41598-018-31017-0

**Published:** 2018-08-20

**Authors:** Lutong Cai, Andrey V. Gorbach, Yiwen Wang, Hui Hu, Wei Ding

**Affiliations:** 1 Laboratory of Optical Physics, Institute of Physics, Chinese Academy of Sciences, Beijing, 100190 China; 2 School of Physics, Shandong University, Jinan, 250100 China; 3 Centre for Photonics and Photonic Materials, Department of Physics, University of Bath, Bath, BA2 7AY UK

## Abstract

The inherent trade-off between efficiency and bandwidth of three-wave mixing processes in *χ*_2_ nonlinear waveguides is the major impediment for scaling down many well-established frequency conversion schemes onto the level of integrated photonic circuit. Here, we show that hybridization between modes of a silica microfiber and a LiNbO_3_ nanowaveguide, amalgamated with laminar *χ*_2_ patterning, offers an elegant approach for engineering broadband phase matching and high efficiency of three-wave mixing processes in an ultra-compact and natively fiber-integrated setup. We demonstrate exceptionally high normalized second harmonic generation (SHG) efficiency of up to *η*_*nor*_ ≈ 460% W^−1^ cm^−2^, combined with a large phase matching bandwidth of Δ*λ* ≈ 100 nm (bandwidth-length product of Δ*λ · L* ≈ 5 *μ*m^2^) near the telecom bands, and extraordinary adjustment flexibility.

## Introduction

Advancement of nano-scale and integrated optical components has always been in the mainstream of research in photonics. While the majority of studies to date are focused on silicon^1,2^, silicon nitride^3^, aluminum nitride^4^, and III-V compound semiconductor platforms^5,6^ for nano-photonic circuits, recent rapid development of the full wafer technology for single-crystalline LiNbO_3_ on insulator (LNOI) thin films^7^ introduces a strong competitor. Lithium niobate (LN), also called the “silicon of photonics (nonlinear optics)” for its outstanding and balanced optical, ferroelectric and electro-optical properties^8^, has been widely recognized as a versatile material for integrated optics^9^. In particular, its broad transparency range (0.4–5.0 *μ*m) combined with the strong second-order (*χ*_2_) nonlinearity are essential to enable three-wave mixing processes. Such processes play important roles in many frequency conversion applications, including second harmonic, sum- and difference-frequency generation. Recently, spontaneous down-conversion (SPDC) in *χ*_2_ nonlinear crystals and waveguides became recognized as one of the key platforms for development of sources of indistinguishable single photons and correlated/entangled photon pairs, that can operate reliably at room temperature and ambient conditions^10,11^. A possibility to substantially reduce the footprint of such devices, offered by the emergence of LNOI nano-waveguides, could trigger a revolutionary advancement in design of functional ultra-compact quantum photonic components and circuits, including ultra-compact photon sources based on spatial multiplexing schemes^12^. However, such devices require a convenient and compact nonlinear waveguide platform that offers the important combination of efficient and broadband *χ*_2_ functionality, low losses, including out-coupling losses to fiber-optic systems, and adjustability.

Being inherently parametric, three-wave mixing processes critically rely on the phase matching condition: while energy conservation establishes the relationship between frequencies of the three interacting waves *ω*_1_ = *ω*_2_ + *ω*_3_, conservation of momentum requires matching of propagation constants *β*(*ω*_1_) = *β*(*ω*_2_) + *β*(*ω*_3_). Due to material dispersion the latter condition is generally not possible to satisfy for plane waves in a bulk medium. Conventional approaches, involving manipulations with input fields (e.g. polarization and angle of incidence) and material structure (e.g. periodic poling), usually require a compromise between the efficiency, bandwidth, and overall size and complexity of the setup.

In waveguides with relatively small core-to-cladding refractive index contrasts Δ*n*_*core*_/*n* ≪ 1 (weak guidance), such as LN micro-waveguides^13^, the material dominated dispersion can be effectively balanced by a periodic poling (PPLN). This quasi-phase matching (QPM) scheme allows highly efficient three-wave mixing processes (normalized SHG efficiency of up to 150% W^−1^ cm^−2^ in the 1550 nm telecom band^13^), and is generally considered as the primary platform for *χ*_2_ waveguides. Recently, periodic poling techniques have been successfully adopted to LNOI films^14^ and nano-waveguides^7,15^. Thanks to tighter light confinement (and hence stronger effective nonlinearities) and substantially reduced poling period, such PPLNOI waveguides can potentially offer an order of magnitude increase of efficiency in a smaller footprint device, compared to PPLN (theoretically estimated value of 1600% W^−1^ cm^−2 ^^15^). In practice, the measured normalized SHG efficiencies in PPLNOI appear to be more modest (up to 160% W^−1^ cm^−2 ^^15^, i.e. an order of magnitude below theoretical estimations, and comparable to best PPLN results) due to non-uniformity of periodic poling.

Taking aside technical challenges associated with periodical structuring in LNOI nano-waveguides^15,16^, the fixed relationship between the longitudinal period and the momentum mismatch for a particular combination of interacting frequencies represents a major generic constraint of QPM scheme. In particular, this leaves little room for any adjustments of individual waveguides integrated in a circuit. The conventional method of temperature or external DC electric field control is not suitable for high-density on-chip integration due to the lack of sufficient resolution. Adiabatic variation of period^17^ and other types of chirping of QPM gratings^18,19^ can be implemented to expand the bandwidth, however, it adds to the technical complexity, and ultimately production costs, of the setup and requires much longer waveguides. Apart from the practical issues due to incongruity with compact design and dense on-chip integration, longer waveguides suffer from stronger walk-offs between interacting harmonics due to group velocity mismatch (GVM). This limits the overall efficiency and bandwidth of three-wave mixing processes, in particular it induces temporal distinguishability (“timing jitter”) and associated visibility degradation of SPDC generated photon pairs^20^. Utilizing the specific dispersion of LNOI thin films, it is possible to reduce GVM for some combinations of interacting harmonics^14^. However, this method requires fine-tuning of LNOI film thickness^14^, which restrains adjustability of individual waveguides produced from a single wafer.

In strongly-guiding LNOI waveguides $({\rm{\Delta }}{n}_{core}/n\gtrsim 1)$, geometrical dispersion is significant and can be utilized to counter-act material dispersion^16,21,22^. Thus modal phase matching (MPM) between modes of different orders becomes a viable alternative to QPM scheme. However, the appreciable modal dispersion and involvement of higher-order modes impose considerable fundamental constraints on both the bandwidth and efficiency of three-wave mixing processes^22^.

In this work, we demonstrate that mode hybridization between a silica microfiber (MF) and LN nano-waveguide represents a very effective method of dispersion management, which allows to achieve simultaneous broadband phase and group velocity matching in a desired wavelength range. We also show that laminar nonlinearity patterning, by virtue of an embedded proton exchange (PE) layer, represents a distinctive way to break the fundamental bottleneck of poor modal overlap in conventional MPM scheme. By introducing a novel hybrid MF-LNOI architecture, we illustrate how the combination of the above two powerful dispersion and nonlinearity engineering tools helps to achieve highly efficient and broadband three-wave mixing processes in a ultra-compact and natively fiber-integrated architecture, that allows high degree of adjustability. In our proof-of-concept SHG experiments we achieve unprecedentedly high conversion efficiencies of up to *η*_*nor*_ ~ 460% W^−1^cm^−2^ with a bandwidth of up to Δ*λ* ~ 100 nm (the bandwidth-length product of up to 5 *μ*m^2^). These results bring the traditionally low-efficient MPM scheme back into competition with the well-established QPM scheme.

## Theory and design

### Bandwidth-efficiency trade-off and dispersion engineering

For SHG process in a waveguide of length *L* pumped by a CW source of power *P*_*F*_, the generated signal power is given by^23^:

1 $${P}_{SH}\propto {({P}_{F}L{\rho }_{2})}^{2}\cdot {{\rm{s}}{\rm{i}}{\rm{n}}{\rm{c}}}^{2}({\rm{\Delta }}\beta L/2),$$

where *ρ*_2_ is the waveguide second-order nonlinear coefficient, pump depletion is neglected under assumption ${L}^{2}{P}_{F}{\rho }_{2}^{2}\ll 1$ (i.e. short waveguide), and Δ*β*(*λ*_*F*_) = 2*β*_*F*_ − *β*_*SH*_ = 4*π*(*n*_*F*_ − *n*_*SH*_)/*λ*_*F*_ is the propagation constant mismatch between the modes at fundamental (*ω*_2_ = *ω*_3_ = *ω*_*F*_) and second harmonic (SH) (*ω*_1_ = *ω*_*SH*_ = 2*ω*_*F*_) frequencies. For efficient interaction, the argument of the sinc function in the above formula must be small: $|{\rm{\Delta }}\beta |L/2\ll \pi$. Hence the inherent trade-off between bandwidth and efficiency of *χ*_2_ processes in nonlinear waveguides becomes apparent: while efficiency grows with the waveguide length as ~*L*^2^, the bandwidth, implicitly determined via Δ*β*(*λ*_*F*_), generally narrows.

Since sinc^2^(0.44*π*) ≈ 0.5, the bandwidth (FWHM) of the function sinc^2^(Δ*βL*/2) in Eq. (1) around a particular wavelength of phase matching [*λ*_*F*0_, Δ*β*(*λ*_*F*0_) = 0] is determined by the roots ${\lambda }_{F}^{\mathrm{(1)}} < {\lambda }_{F0} < {\lambda }_{F}^{\mathrm{(2)}}$ of the following equation:

2 $$\frac{2{\rm{\Delta }}{n}_{eff}({\lambda }_{F})}{{\lambda }_{F}}=\pm \,\frac{0.44}{L},$$

where Δ*n*_*eff*_ (*λ*_*F*_) = *n*_*F*_(*λ*_*F*_) − *n*_*SH*_(*λ*_*F*_/2). For long waveguides with $L\gg {\lambda }_{F}$, the roots of the above equation converge, ${\lambda }_{F}^{\mathrm{(1,2)}}\to {\lambda }_{F0}$, and the bandwidth shrinks. In a vicinity of *λ*_*F*0_, the l.h.s. of Eq. (2) can be approximated as: $2{\rm{\Delta }}{n}_{eff}/{\lambda }_{F}\approx 2({n^{\prime} }_{F}-{n^{\prime} }_{SH})\cdot {\lambda }_{F0}^{-1}\cdot ({\lambda }_{F}-{\lambda }_{F0})$ with ${n^{\prime} }_{F,SH}=d{n}_{F,SH}/d{\lambda }_{F}$. Accordingly, the bandwidth ${\rm{\Delta }}\lambda =$ ${\lambda }_{F}^{\mathrm{(1)}}-{\lambda }_{F}^{\mathrm{(2)}}$ is inversely proportional to the waveguide length *L*, and the product Δ*λ*⋅*L* does not depend on *L*:

3 $${\rm{\Delta }}\lambda \cdot L\approx \frac{0.44{\lambda }_{F0}}{{n^{\prime} }_{F}-{n^{\prime} }_{SH}}.$$

The single-core geometry of a LNOI waveguide offers limited degrees of freedom for dispersion engineering: adjusting height and width of the waveguide, one can achieve phase matching between different pairs of modes at a desired wavelength, but there is no control over the gradient mismatch. For LNOI waveguides, the latter can be as high as $({n^{\prime} }_{F}-{n^{\prime} }_{SH}) \sim 0.5\,\mu {{\rm{m}}}^{-1}$, limiting the bandwidth-length product to ${\rm{\Delta }}\lambda \cdot L\lesssim 1.5\,\mu {{\rm{m}}}^{2}$ when using *λ*_*F*_ = 1.5 *μ*m. On the other hand, in PPLN and PPLNOI waveguides^13,15^, by using QPM, this bandwidth-length product can reach ${\rm{\Delta }}\lambda \cdot L \sim 10\,\mu {{\rm{m}}}^{2}$.

To resolve the intrinsic bandwidth-efficiency conflict in MPM scheme, an advanced control over waveguide dispersion is required, that would allow simultaneous and independent management of effective indexes and their gradients. In this work we demonstrate how the well-known mode hybridization mechanism can be effectively exploited for this purpose.

**Figure 1:**
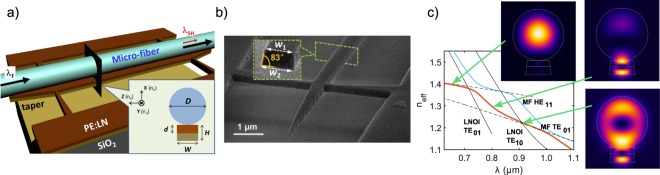
Fiber-integrated LNOI waveguide, MF-LNOI: (**a**) Schematic view of the structure on an X-cut LNOI wafer. A proton exchanged layer of adjustable thickness *d* is introduced to the upper part of the 300 nm lithium niobate thin film (PE:LN). LNOI nanowaveguide with linearly tapered in- and out-coupling sections is held by suspending arms, and a MF of diameter *D* is attached on top; (**b**) Scanning electron microscopy (SEM) image of a waveguide, showing the in-coupling taper and suspending arms. The waveguide has a trapezoid cross-section with the base angle of ~83°, and the top and bottom widths of *w*_1_ and *w*_2_, respectively; (**c**) Dispersion curves of two hybridized MF-LNOI modes [effective index *n*_*eff*_ = *βλ*/(2*π*), the blue and the red lines]. The geometric parameters are: *w*_1_ = 474 nm, *w*_2_ = 544 nm, *d* = 130 nm, and microfiber diameter *D* = 1.1 *μ*m. Each hybridized mode can be phase-matched with a fundamental pump mode in a broad wavelength range (the two cases in our experiment). Insets show variation of one mode profile (intensity) with wavelength. Thin dashed and solid curves show dispersions of isolated MF and LNOI modes, respectively, whose intersections indicate the places where anti-crossings of hybridized modes occur.

**Figure 2:**
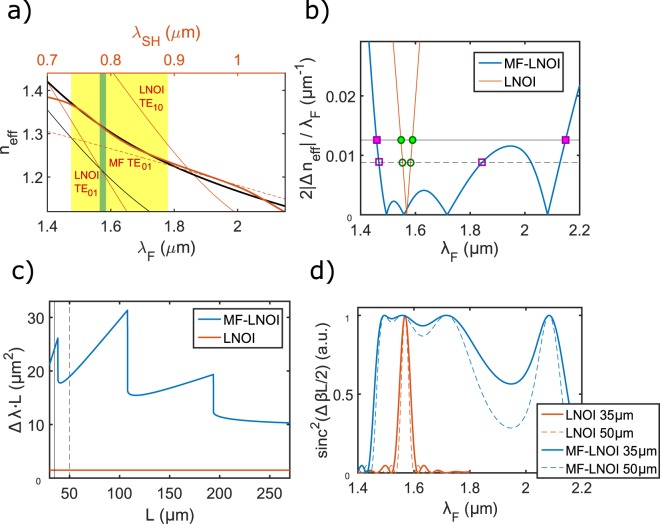
The effect of mode hybridization on phase matching: (**a**) Effective modal indices at the fundamental (black, bottom axis) and respective SH (red, top axis) wavelengths in the isolated LNOI waveguide with *w*_1_ = 474 nm, *w*_2_ = 544 nm (thin curves), and in the MF-LNOI structure (bold curves) with MF diameter *D* = 1.1  *μ*m [same geometry as in Fig. 1(c)]. In the MF-LNOI structure the SH mode dispersion is affected by a sequence of hybridizations between *TE*_01_ mode of MF (dashed curve) and *TE*_01_, *TE*_10_ modes of LNOI waveguide [cf. Fig. 1(c)]. Shaded areas indicate phase-matching bandwidths [FWHM of sinc^2^(Δ*βL*/2)] for the isolated LNOI waveguide (dark green shading) and MF-LNOI structure (light yellow shading) of length *L* = 50 *μ*m; (**b**) Graphical solution of the bandwidth condition in Eq. (2). L.h.s. of Eq. (2) is plotted with bold (thin) curves for MF-LNOI (LNOI) structure. Two horizontal levels correspond to r.h.s. of Eq. (2) with *L* = 35 *μ*m (solid) and *L* = 50 *μ*m (dashed); (**c**) Bandwidth-length product calculated for the phase-matching point near *λ*_*F*_ = 1.55 *μ*m in LNOI and MF-LNOI structures. A stepwise increase is seen for the latter structure due to merging of peaks of the phase-matching function (at *λ*_*F*_ ≈ 1.72 *μ*m and *λ*_*F*_ ≈ 2.1 *μ*m); (**d**) Phase-matching function sinc^2^(Δ*βL*/2) for LNOI and MF-LNOI structures.

The central element of our design is a vertical stack of a silica microfiber (MF) and a free-standing LNOI waveguide supported by suspending arms, as shown in Fig. 1(a,b). The modest refractive index difference between silica and LN, and the suspended geometry of LNOI waveguide, make it possible to bring propagation constants of guided modes of the MF and LNOI waveguide close together. The resulting mode hybridization, featured by a series of anti-crossing points, has a strong impact on the dispersion of the MF-LNOI structure, see Fig. 1(c). Proper adjustments of geometrical parameters can bring suitable modifications to the overall dispersion, as the positions of anti-crossing points shift. Having three parameters available to tailor (fiber diameter, waveguide height and width), this represents a powerful approach to dispersion (and its gradient) engineering, and give us enough flexibility to fine-tune the structure and compensate for manufacturing inaccuracies. Earlier we demonstrated theoretically how by tuning the MF diameter and LNOI waveguide size, phase matching between different sets of modes and in a desired wavelength range can be arranged^24^. In Fig. 2(a) such engineered phase matchings are illustrated as crossings between *n*_*F*_(*λ*_*F*_) and *n*_*SH*_(*λ*_*F*_/2) curves for LNOI waveguide (single crossing at around *λ*_*F*0_ ≈ 1.55 *μ*m) and MF-LNOI hybrid structure (altogether four crossings in the interval 1.45 *μ*m < *λ*_*F*_ < 1.9 *μ*m). What was overlooked in our previous work^24^ is the advantage of strong modification of the dispersion of hybridized modes for expanding the phase matching bandwidth. Indeed, hybridization offers an additional “lever” to simultaneously minimize phase (*n*_*F*_ − *n*_*SH*_) and its gradient $({n^{\prime} }_{F}-{n^{\prime} }_{SH})$ mismatches. Since gradients of the interacting MF and LNOI modes differ considerably, by effectively “averaging” the gradients in hybridized MF-LNOI modes, which happens near anti-crossing points, one can strongly tailor the gradient of the corresponding mode within broad wavelength ranges, see Fig. 2(a). This represents a completely unexploited paradigm for nano-waveguide dispersion engineering.

Furthermore, multiple anti-crossings between a MF mode and several LNOI modes of different orders result in a nonlinear relationship of Δ*n*(*λ*_*F*_), cf. Figs 1(c) and 2(a,b). This breaks the above described inverse proportionality of bandwidth and waveguide length, typical for conventional waveguides. In Fig. 2(b) the condition determining FWHM of the sinc^2^(Δ*βL*/2) function, i.e. Eq. (2), is presented graphically, while in Fig. 2(c) we plot the product Δ*λ*⋅*L* as a function of the waveguide length *L*. In conventional LNOI waveguide the linear characteristic of Δ*n*(*λ*_*F*_) results in an *L*-independent product of Δ*λ*⋅*L*. In long MF-LNOI waveguide [e.g. *L* > 200 *μ*m in Fig. 2(c)], the phase-matching function represents a series of isolated peaks, each demonstrating a similar linear relationship of Δ*n*(*λ*_*F*_) as in conventional waveguides. In this limit, the product Δ*λ*⋅*L* approaches a constant value (~10 *μ*m^2^), similar to PPLN and PPLNOI waveguides. Remarkably, due to substantial reduction of the gradient mismatch between hybridized MF-LNOI modes, this limiting value has been nearly one order of magnitude higher than in typical LNOI waveguides. Yet the most appealing advantage of MF-LNOI geometry is in the ability to depart from the conventional constant trend of Δ*λ*⋅*L* when the waveguide length becomes small enough. In this regime, the nonlinear nature of Δ*n*(*λ*_*F*_) becomes apparent, and the phase-matching function differs considerably from the standard sinc-square shape [see the curves in Fig. 2(d) for LNOI and MF-LNOI structures]. As illustrated in Fig. 2(c,d), for *L* < 200 *μ*m adjacent peaks of the phase-matching function start to merge, resulting in a series of step-wise increases of bandwidth (and bandwidth-length product). The nonlinear relationship of Δ*n*(*λ*_*F*_) thereby provides a new mechanism to bypass fundamental bandwidth-efficiency conflict without sacrifice of size compactness.

### Modal overlap and nonlinearity engineering

The effective nonlinear coefficient *ρ*_2_ is determined by modal overlap^23,25^:

4 $${\rho }_{2}=\frac{\pi c{\varepsilon }_{0}}{2{\lambda }_{F}\sqrt{{N}_{SH}}{N}_{F}}{\iint }_{WG}{\overrightarrow{{\bf{e}}}}_{SH}\cdot ({\hat{\chi }}_{2}\vdots {\overrightarrow{{\bf{e}}}}_{F}^{2})dA.$$

Here ${\overrightarrow{{\bf{e}}}}_{F}$ and ${\overrightarrow{{\bf{e}}}}_{SH}$ are electric field profiles of the fundamental and SH modes, ${\hat{\chi }}_{2}$ is the LN second-order nonlinear tensor^26^, and the integral is taken over the waveguide cross-section, ${N}_{k}=\mathrm{(1}/\mathrm{4)}\iint ({\overrightarrow{{\bf{e}}}}_{k}\times {\overrightarrow{{\bf{h}}}}_{k}^{\ast }+{\overrightarrow{{\bf{e}}}}_{k}^{\ast }\times {\overrightarrow{{\bf{h}}}}_{k})\cdot \hat{y}dA$, (*k* = *F*,*SH*), are normalization factors (total power carried by each guided mode in the propagation direction, *y*). In QPM scheme interaction can happen between modes of the same order (typically, lowest order modes), while the phase mismatch is canceled out by introducing longitudinal periods. This guarantees a strong modal overlap, and hence highly efficient three-wave mixing. However, in MPM scheme the two modes have different symmetries, and the resulting overlap is generally weak^22,27^ - which is a major pitfall of this scheme. While the abolishment of longitudinal modulation makes MPM scheme particularly attractive for design and fabrication of integrated photonic components, poor modal overlap requires long waveguides for efficient interactions, and thus revokes any advantages over QPM.

Additionally, both the nonlinearity (*ρ*_2_) and dispersion (Δ*β*) characteristics are governed by the same waveguide geometry, and hence are inherently linked. This represents another fundamental problem of *χ*_2_ waveguides: an independent control of *ρ*_2_ and Δ*β* is usually not possible. Removing this constrain would substantially enrich design capabilities for engineering of broadband and efficient three-wave mixing processes.

**Figure 3:**
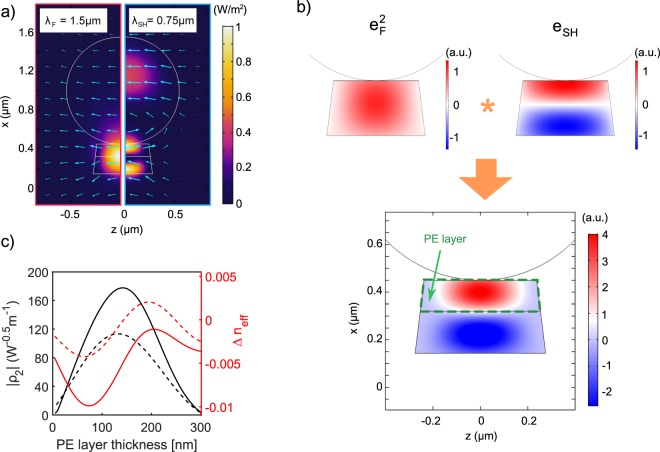
The effect of *χ*_2_ patterning on modal overlap: (**a**) Intensity profiles of the fundamental and SH modes at *λ*_*F*_ = 1.5 *μ*m (*λ*_*SH*_ = 0.75 *μ*m) for the structure as in Fig. 1(c). Arrows indicate in-plane orientation of electric field; (**b**) Different phase structures of the dominant electric field component (*e*_*x*_) in the two interacting modes are illustrated in the top row, and the corresponding overlap function as integrand in Eq. (4) is shown in the bottom. Introducing PE layer of thickness 130 nm, the integrand is suppressed inside the indicated dashed frame; (**c**) Nonlinear coefficient and effective index mismatch Δ*n*_*eff*_ = *n*_*F*_ − *n*_*SH*_ as functions of PE layer thickness *d* for *λ*_*F*_ = 1.5 *μ*m (solid curves) and *λ*_*F*_ = 1.6 *μ*m (dashed curves).

Here we demonstrate how laminar nano-structuring offers an elegant solution to the problem of poor modal overlap, and helps to achieve nearly independent control of nonlinearity and dispersion of a waveguide. The second important element of our architecture is the nonlinearity patterning via introduction of a shallow PE layer. This enables us to control the modal overlap by enforcing the transverse profile of the material tensor, and thus to engineer the effective nonlinearity of the whole structure. In Fig. 3(a) profiles of a pair of phase-matched modes are illustrated. Despite having a good overlap of intensities in LN, due to the different phase structure of these modes, the integrand in Eq. (4) changes sign across the waveguide as shown in Fig. 3(b). By introducing a shallow PE layer^28^, we eliminate *χ*_2_ nonlinearity of LN in the corresponding upper part of the waveguide^29,30^. Interestingly, an appropriate local suppression of material nonlinearity helps to enhance the effective waveguide nonlinearity. By inhibiting one of the lobes of the integrand function, we boost the effective nonlinear coefficient of the whole waveguide by almost two orders of magnitude, see Fig. 3(c), reaching the level of best performing PPLN and PPLNOI waveguides. Thus laminar nonlinearity patterning technique, reconciled with field distributions of interacting modes, resolves the fundamental problem of poor modal overlap in MPM scheme, and revives the competition with QPM.

Furthermore, PE process is known to introduce less than 5% variations to the refractive index of LN (see Supplementary Fig. S1)^28,30^. Insertion of a shallow PE layer hence brings only minor changes to linear properties of the waveguide, i.e. field profiles and mode dispersion. Such partial PE of adjustable depth represents a powerful technique to control nonlinearity, which does not bring a notable disturbance to the phase matching, see the plots of Δ*n*_*eff*_ in Fig. 3(c). This independent nonlinearity engineering is a unique feature of our platform, which can facilitate nonlinear waveguide design.

## Experiment

**Figure 4:**
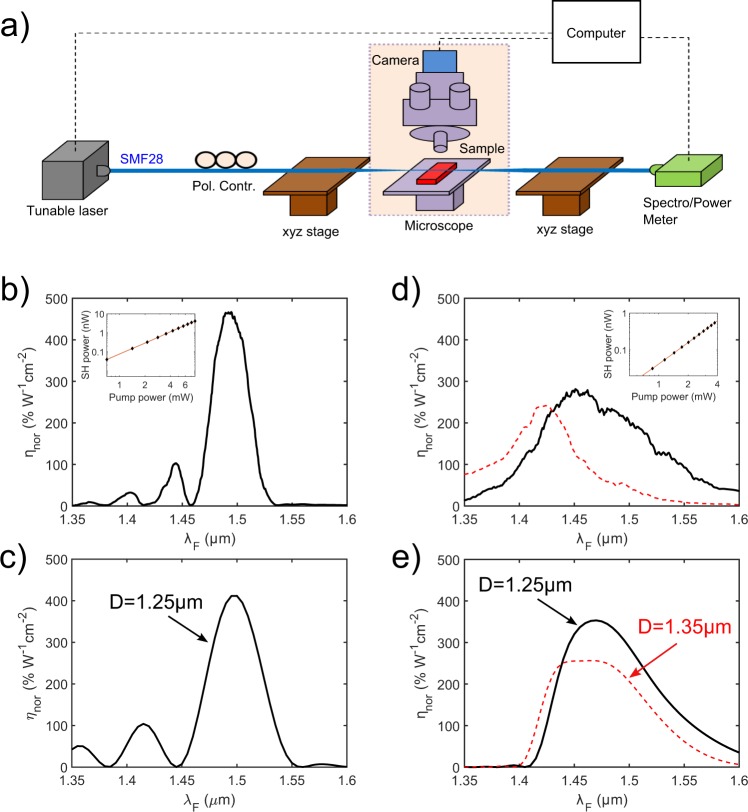
SHG in MF-LNOI structures: (**a**) Schematic of experimental setup; (**b**–**e**) Normalized SHG efficiency ${\eta }_{nor}={P}_{SH}/({P}_{F}^{2}{L}^{2})$ in structures of length *L* = 50 *μ*m and different waveguide widths: SEM measured widths of (*w*_1_ = 530 nm, *w*_2_ = 600 nm) (sample A, left column) and (*w*_1_ = 460 nm, *w*_2_ = 530 nm) (sample B, right column). Data are obtained from experimental measurements (**b**,**d**) and numerical simulations (**c**,**e**). Insets in (**b**,**d**) show dependence of SH power versus pump power (raw data) in log-log scale for *λ*_*F*_ = 1.495 *μ*m and *λ*_*F*_ = 1.450 *μ*m, respectively. Linear fit with slopes 1.96 (**b**) and 1.94 (**d**) (red curves) confirm the quadratic dependence characteristic of SHG process. In numerical simulations the trapezoid waveguide shape was adopted with top and bottom widths as (*w*_1_ = 536 nm, *w*_2_ = 606 nm) (sample A) and (*w*_1_ = 474 nm, *w*_2_ = 544 nm) (sample B). The thicknesses of the LN film and the upper PE layer are respectively 300 nm and 130 nm. Two data sets in (**d**,**e**) (solid and dashed curves) are acquired from different sections of MF attached to the LNOI waveguide in experiment, and for different MF diameters in simulations.

We prepared two LNOI waveguides of the same length *L* = 50 *μ*m and widths of ~565 nm (sample A) and ~495 nm (sample B). From numerical simulations and experiments we found that MF diameter *D* ~ 1.25 *μ*m gives the broadest phase-matching, while PE layer of thickness ~130 nm maximizes the nonlinear coefficient in both samples. In experiment we mounted different sections of one MF with a few centimeter length on the top of waveguides to adjust the diameter parameter *D*, see Fig. 4(a).

To obtain conversion efficiency, we first measured input power by monitoring transmitted pump through isolated (not in contact with LNOI) MF. Then, we attached the MF to the LNOI waveguide and measured generated SH signal. The raw data of output SH power *vs* input pump power are plotted in the insets of Fig. 4(b,d) for samples A and B when pumped at *λ*_*F*_ = 1.495 *μ*m and *λ*_*F*_ = 1.450 *μ*m, respectively. From 7.8 mW (5.75 mW) pump power in sample A(B) we measured ~4.1 nW (~1.37 nW) SH light. In order to estimate the pump power coupled into the MF-LNOI structure and the SH power at the output end of the waveguide, and then to calculate the conversion efficiency, we calibrated our measurements by taking into account simulated in- and out-coupling losses for pump and SH lights (see Supplementary Fig. S2). In both samples, we obtained exceptionally high normalized efficiencies, reaching the peak values to 460% W^−1^ cm^−2^ (290% W^−1^ cm^−2^) in sample A(B), see Fig. 4(b,d). This is more than six orders of magnitude higher than reported results of SHG in fibers^31^, and several times higher than in PPLN waveguides^13,15^. As illustrated in Fig. 4(c,e), our experimental measurements are in good agreement with numerical simulations. Remarkably, the high efficiencies are combined with large bandwidths of 38 nm in sample A and 108 nm in sample B. The bandwidth-length products are 1.9 *μ*m^2^ and 5.4 *μ*m^2^, respectively. The apparent reduction of this product, compared to theoretical estimates in Fig. 2(c), is due to the dispersion of nonlinear coefficient, *ρ*_2_(*λ*_*F*_), which introduces additional restraint to the bandwidth according to Eq. (1).

We emphasize that MF and LNOI are kept together and aligned symmetrically by virtue of van der Waals and electrostatic attraction. The adjustability of mode dispersion by varying geometrical parameters of the structure, and the possibility to detach and re-attach MF at different sections along the fiber taper, provide unique flexibility in fine tuning bandwidth and efficiency of generic three-wave mixing processes. By adjusting MF position in sample B we observe a considerable spectrum shift of the peak SHG efficiency, as shown in Fig. 4(d) by the dashed curve. Numerical simulation verifies the similar behavior when varying the MF diameter, as shown in Fig. 4(e).

## Discussion and Conclusion

We believe that the consolidation of mode hybridization and nonlinearity patterning by means of laminar nano-structuring represents a novel versatile approach to design of ultra-compact *χ*_2_ nonlinear waveguides. Particularly, it offers advanced tools for comprehensive and independent dispersion and nonlinearity engineering of the structure. In a proof-of-concept experiment, we demonstrate a combination of high normalized efficiency, broad bandwidth (large bandwidth-length product), and high degree of tunability of three-wave mixing processes in a compact and natively fiber-integrated MF-LNOI architecture.

We show how the mode hybridization provides a unique and powerful method to adjust and match index gradients *n*’ = *dn*/*dλ*_*F*_ of interacting harmonics. This helps to break the fundamental trade-off between bandwidth and efficiency, inherent in conventional *χ*_2_ waveguides, and potentially achieve extremely broad bandwidths without a necessary sacrifice of efficiency in compact waveguides. Furthermore, the simultaneously obtained matching of effective indexes and their gradients ensures matching of group velocities of interacting harmonics, *c*/*v*_*g*_ = *n* − *n*’*λ*. The suppression of the walk-off between interacting waves is of essential importance for efficient frequency conversion with pulsed sources and improving visibility of SPDC generated photon pairs.

Also, we demonstrate how nonlinearity patterning resolves the well-known problems of poor modal overlap and lack of independent control of nonlinearity and dispersion in conventional MPM scheme. Interplay between dispersion and nonlinearity is an important and fundamental aspect of all nonlinear optical processes. The comprehensive and independent manipulation of these two characteristics demonstrated in our MF-LNOI platform enables versatile functional waveguide design, and opens new avenues for research in nonlinear and quantum photonics.

The geometry of proposed MF-LNOI structure allows efficient multi-parameter adjustments of the bandwidth and efficiency of three-wave mixing processes. We demonstrated a considerable shift of SHG peak wavelength by changing the MF diameter. Compared to conventional methods of varying temperature or external electric field, such geometrical adjustment provides much higher resolution and accuracy, which is essential for fine-tuning of densely integrated photonic devices. Furthermore, appropriate adjustments of the MF diameter can be used to compensate for manufacturing imperfections. Following the fine-tuning procedure, a firm fixing of MF on LNOI waveguide could be realized e.g. with the help of optically transparent adhesives.

Last but not least, inefficient coupling to conventional fiber optic systems causes significant losses^7^ and restrains functionality of nano-waveguides, especially for photon sources and quantum information applications. Here, MF-LNOI platform can offer an outstanding alternative to conventional waveguide. Thanks to the exceptionally weak material absorption and atomic level surface flatness, silica MF exhibit excellent light guidance in a broad spectral range^32,33^. Introducing in- and out-coupling tapering sections of LNOI waveguide, as shown in Fig. 1(a,b), we can utilize adiabatic mode conversion to optimize light coupling. When used in combination with the taper transition from MF to conventional fiber, a nearly lossless integration of the MF-LNOI nano-structure with fiber optics can ultimately be arranged.

The combination of all the above features is very advantageous for the development of integrated nonlinear and quantum photonic circuits. For such applications, we believe the platform demonstrated in this work can offer a superior alternative to other conventional *χ*_2_ waveguides.

## Methods

### Sample fabrication and characterization

An X-cut LNOI wafer with a ~300 nm-thick LN film bonded on a SiO_2_/LN substrate was used. The wafer was immersed in a molten benzoic acid at 200 °C for 3 minutes. The exchange of *H*^+^ ions (proton) in the melt and *Li*^+^ ions in the LN thin film resulted in a shallow layer of step-like *β*_*i*_-phase PE:LN with the depth of roughly half of the total LN thickness measured by focused ion beam (FIB) milling cut. After PE we did not carried out annealing in order to preclude further proton diffusion and keep *χ*_2_ in the PE:LN layer nearly zero. Then, 100 nm-thick chromium (Cr) was deposited onto the surface to serve as a conductive and protective coating in the milling process. FIB milling within one write-field was applied to fabricate waveguides of designed widths and length (50 *μ*m) along the Y axis of the crystal. According to our previous calculation^24^, the waveguide width non-uniformity will not affect SHG in such a distance. An acceleration voltage of 30 kV and a beam current of 100 pA ensure smooth and nearly vertical sidewalls of the waveguides. The milling process introduces a sidewall angle of ~83°, as measured in SEM image. We adapted the corresponding cross-section in our modeling. On both ends of each waveguide linear tapers and suspending arms were added to facilitate light coupling and mechanical strength, respectively, as shown in Fig. 1(b). Finally, the Cr coating was removed by Cr etchant, and the *SiO*_2_ layer (~2 *μ*m thick) beneath the LNOI waveguide was wet etched by hydrofluoric acid. After fabrication the free-standing LNOI waveguides can withstand chemical cleaning and preserve good qualities of optical and mechanical properties over more than one year.

A silica MF was fabricated from conventional optical fiber (Corning SMF28) by standard heat-and-pull technique^34^. Its insertion loss was measured to be less than 0.1 dB at both fundamental and SH wavelengths. It can be attached to and detached from the LNOI waveguide repeatedly, and can slip along the waveguide smoothly (see Supplementary video), thus allowing us to fine-tune dispersion of the MF-LNOI structure.

By measuring the output powers at 1500 nm from fiber pigtail with and without the LNOI waveguide (sample A) attaching to the MF, we also attain the in/out-coupling loss at this wavelength to be ~1.1 dB, agreeing well with the simulated result of 1.0 dB. This insertion loss is caused by a linear taper of length 12.5 *μ*m and two 0.3 *μ*m-wide suspending arms. FDTD simulations give the in/out-coupling losses at other wavelengths and modes. Detailed information on the measurements and simulations can be found in Supplementary Sec. S2.

### Modeling

Propagation constants and field profiles of fundamental and SH modes, which are needed in calculation of Δ*β* and *ρ*_2_, were obtained with the help of COMSOL Multiphysics software. In simulations we adopted the trapezoid shape of LNOI waveguide with a PE layer of variable thickness, as shown in Fig. 3(b). We used available and well established in literature material dispersions of fused silica^23^ and LN^26^.

According to our previous work^28^, for shallow depths of PE channel, the lateral diffusion can be ignored, and so we applied the step changes of ordinary and extraordinary LN refractive indeces, Δ*n*_*o*_ and Δ*n*_*e*_, inside the PE layer. We measured the corresponding changes of LN refractive index by prism coupling method at wavelengths of 633 nm and 1539 nm (see Supplementary Fig. S1 for details). Our results of Δ*n*_*e*_ are in good agreement with the empirical Sellmeier equation reported in ref.^35^:

5 $${\rm{\Delta }}{n}_{e}=\sqrt{0.00743+\frac{0.00264\mu {m}^{2}}{{\lambda }^{2}-{\mathrm{(0.336}\mu m)}^{2}}}.$$

For the ordinary refractive index, we adopted a constant value of Δ*n*_*o*_ = −0.06035.

We believe that the uncertainty of the refractive index profile across the PE layer is the major cause of discrepancies between theoretical and experimental SHG efficiency data reported in Fig. 4.

For calculations of the nonlinear coefficient *ρ*_2_ we used the reduced second order nonlinear tensor $\hat{d}$ (${\hat{\chi }}_{2}$ = 2$\hat{d}$) with the following structure^26^:

6 $$\hat{d}=[\begin{array}{cccccc}0 & 0 & 0 & 0 & {d}_{31} & -{d}_{22}\\ -{d}_{22} & {d}_{22} & 0 & {d}_{31} & 0 & 0\\ {d}_{31} & {d}_{31} & {d}_{33} & 0 & 0 & 0\end{array}],$$

where *d*_22_ = 2 pm/V, *d*_31_ = 5 pm/V, and *d*_33_ = 19 pm/V^36^. All components of $\hat{d}$ were set to zero inside the PE layer^29,30^.

### SHG efficiency measurements

The schematic of our experimental setup is shown in Fig. 4(a). A tapered MF was fixed at and precisely positioned by two xyz-stages. An optical microscope with a CCD camera was used to monitor MF and LNOI waveguide. At one end of the MF, a tunable laser (Santec TSL210) launched light into the sample through an in-line polarization controller. At the other end, a highly-sensitive visible spectrometer (Ocean Optics QE65000) and a power meter were used to measure the SH wavelength and power. Since the spectrometer only responds in the visible, we had not used filter to remove the pump light. Before measurement, the spectrometer was carefully calibrated with the power meter. The normalized SHG efficiencies inside the MF-LNOI structures were calculated by taking into account the in/out-coupling losses, as illustrated in Supplementary Fig. S2.

## Electronic supplementary material

Supplementary information

video

## References

[1] Simply silicon. *Nat. Photonics* **4**, 491 (2010).

[2] Leuthold J, Koos C, Freude W (2010). Nonlinear silicon photonics. Nat. Photonics.

[3] Moss DJ, Morandotti R, Gaeta AL, Lipson M (2013). New CMOS-compatible platforms based on silicon nitride and Hydex for nonlinear optics. Nat. Photonics.

[4] Xiong C, Pernice WHP, Tang HX (2012). Low-Loss, Silicon integrated, Aluminum Nitride photonic circuits and their use for electro-optic signal processing. Nano Lett..

[5] Huang Y, Duan X, Lieber CM (2005). Nanowires for integrated multicolor nanophotonics. Small.

[6] Yan R, Gargas D, Yang P (2009). Nanowire photonics. Nat. Photonics.

[7] Poberaj G, Hu H, Sohler W, Günter P (2012). Lithium niobate on insulator (LNOI) for micro-photonic devices. Laser & Photonics Rev..

[8] Manzo, M., Laurell, F., Pasiskevicius, V. & Gallo, K. Lithium Niobate: The Silicon of Photonics! 421–422 (Springer, Dordrecht, 2013).

[9] Lawrence M (1993). Lithium niobate integrated optics. Reports on Prog. Phys..

[10] Hong CK, Mandel L (1986). Experimental realization of a localized one-photon state. Phys. Rev. Lett..

[11] Eisaman MD, Fan J, Migdall A, Polyakov SV (2011). Invited Review Article: Single-photon sources and detectors. Rev. Sci. Instruments.

[12] Collins MJ (2013). Integrated spatial multiplexing of heralded single photon sources. Nat. Commun..

[13] Parameswaran KR (2002). Highly efficient second-harmonic generation in buried waveguides formed by annealed and reverse proton exchange in periodically poled lithium niobate. Opt. Lett..

[14] Li G, Chen Y, Jiang H, Chen X (2017). Broadband sum-frequency generation using d33 in periodically poled LiNbO_3_ thin film in the telecommunications band. Opt. Lett..

[15] Chang L (2016). Thin film wavelength converters for photonic integrated circuits. Optica.

[16] Wang C (2017). Second harmonic generation in nano-structured thin-film lithium niobate waveguides. Opt. Express.

[17] Suchowski H, Porat G, Arie A (2014). Adiabatic processes in frequency conversion. Laser & Photonics Rev..

[18] Tehranchi A, Kashyap R (2008). Design of novel unapodized and apodized step-chirped quasi-phase matched gratings for broadband frequency converters based on second-harmonic generation. J. Light. Technol..

[19] Bostani A, Tehranchi A, Kashyap R (2017). Super-tunable, broadband up-conversion of a high-power CW laser in an engineered nonlinear crystal. Sci. Rep..

[20] Harada K-i (2011). Indistinguishable photon pair generation using two independent silicon wire waveguides. New J. Phys..

[21] Hu H, Ricken R, Sohler W (2009). Lithium niobate photonic wires. Opt. Express.

[22] Geiss R (2015). Fabrication of nanoscale lithium niobate waveguides for second-harmonic generation. Opt. Lett..

[23] Agrawal, G. P. *Nonlinear Fiber Optics*, 5th edn (Academic Press, 2013).

[24] Gorbach A, Ding W (2015). Microfiber-lithium niobate on insulator hybrid waveguides for efficient and reconfigurable second-order optical nonlinearity on a chip. Photonics.

[25] Boyd, R. W. *Nonlinear Optics*, second edn (Academic Press, 2003).

[26] Dmitriev, V. G., Gurzadian, G. G. & Nikogosian, D. N. *Hanbook of Nonlinear Optical Crystals*, 3rd edn (Springer-Verlag Berlin and Heidelberg GmbH\& Co, Berlin, 1996).

[27] Rao SV, Moutzouris K, Ebrahimzadeh M (2004). Nonlinear frequency conversion in semiconductor optical waveguides using birefringent, modal and quasi-phase-matching techniques. J. Opt. A.

[28] Cai L, Kong R, Wang Y, Hu H (2015). Channel waveguides and y-junctions in x-cut single-crystal lithium niobate thin film. Opt. Express.

[29] Bortz ML, Eyres LA, Fejer MM (1993). Depth profiling of the d33 nonlinear coefficient in annealed proton exchanged LiNbO_3_ waveguides. Appl. Phys. Lett..

[30] Korkishko YN, Fedorov VA, Laurell F (2000). The SHG-response of different phases in proton exchanged lithium niobate waveguides. IEEE J. Sel. Top. Quant..

[31] Canagasabey A (2009). High-average-power second-harmonic generation from periodically poled silica fibers. Opt. Lett..

[32] Tong L (2003). Subwavelength-diameter silica wires for low-loss optical wave guiding. Nature.

[33] Brambilla G (2009). Optical fiber nanowires and microwires: fabrication and applications. Adv. Opt. Photonics.

[34] Birks T, Li Y (1992). The shape of fiber tapers. J. Light. Technol..

[35] Bortz ML, Fejer MM (1991). Annealed proton-exchanged LiNbO_3_ waveguides. Opt. Lett..

[36] Shoji I, Kondo T, Kitamoto A, Shirane M, Ito R (1997). Absolute scale of second-order nonlinear-optical coefficients. J. Opt. Soc. Am. B.

