# Supplementary material for: Highly efficient broadband second harmonic generation mediated by mode hybridization and nonlinearity patterning in compact fiber-integrated lithium niobate nano-waveguides
Source: Sci Rep. 2018 Aug 20;8:12478. doi: 10.1038/s41598-018-31017-0 (PMC6102234; doi:10.1038/s41598-018-31017-0)
Supplement: Supplementary file 1 — Supplementary information [file 41598_2018_31017_MOESM1_ESM.docx]

Supplementary Information

**Highly efficient broadband second harmonic generation mediated by mode hybridization and nonlinearity patterning in compact fiber-integrated lithium niobate nano-waveguides**

Lutong Cai,^1,2^ Andrey V. Gorbach,^3^ Yiwen Wang,^2^ Hui Hu,^2^ and Wei Ding^1,*^

^1^Laboratory of Optical Physics, Institute of Physics, Chinese Academy of Sciences, Beijing 100190, China

^2^School of Physics, Shandong University, Jinan 250100, China

^3^Centre for Photonics and Photonic Materials, Department of Physics, University of Bath, Bath BA2 7AY, UK

^*^Corresponding author: [wding@iphy.ac.cn](mailto:wding@iphy.ac.cn)

**S1. Prism coupling measurement of refractive-index change from LN to PE:LN**

We measured the refractive-index changes of LiNbO_3_ thin film caused by PE treatment. An X-cut LNOI wafer with ~300 nm thin film was fully converted to PE:LN in a molten benzoic acid at 200 ºC for 15 min. We did not perform annealing after PE to ensure the thin film having a zero nonlinear susceptibility. Standard prism coupling measurement [1] was employed and gave ∆n_e_ = 0.1264/0.0966 and ∆n_o_ = -0.0646/-0.0561 at λ = 633/1539 nm, respectively. As shown in Supplementary Fig. S1, the measured extraordinary index changes (∆n_e_) agree well with the Sellmeier equation of bulk as-proton-exchanged LiNbO_3_ crystal reported in Ref. [2]. While, the measured ordinary index changes (∆n_o_), which are also of importance in determining modal indices for a nanowaveguide, do not fit with the results in Ref. [3]. This discrepancy can be attributed to different treatment conditions: in our work and Ref. [2], the PE process was carried out at lower temperatures (200 ºC / 160~220 ºC) for short time durations, in open atmosphere, and by use of pure benzoic acid; while in [3], it was done at 247 ºC for 26 hours, in a sealed ampoule, and by use of benzoic acid diluted with 1% of lithium benzoate. In FDTD simulations (see Fig. 4 in main text), we adopted the Sellmeier equation of ∆n_e_ in Ref. [2] and the average value of the measured ∆n_o_ (the red dotted line in Supplementary Fig. S1).


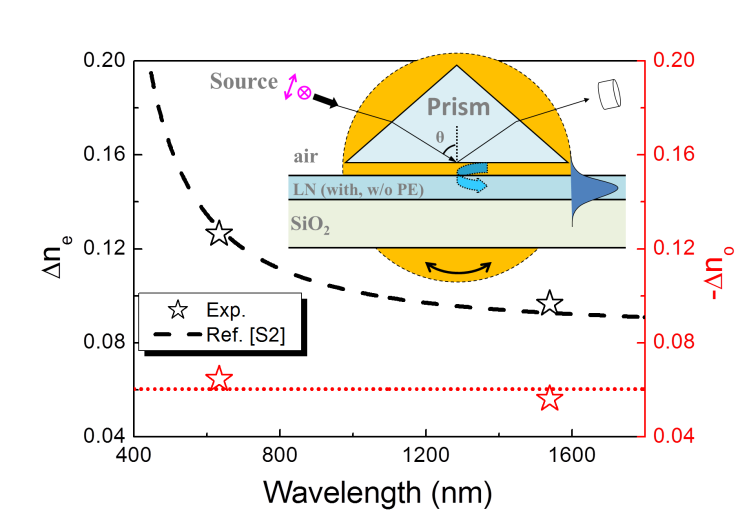


**Supplementary figure S1. Refractive-index changes caused by PE (∆n_e_ and -∆n_o_).** The star points represent the measured results, the dashed line is the Sellmeier equation in Ref. [2] for bulk as-proton-exchanged LiNbO_3_ crystal, and the dotted line is the average value of the measured |∆n_o_|. The inset shows the schematic of the prism coupling measurement setup.

**S2. FDTD simulation of in/out-coupling loss**

Eliminating in/out-coupling losses, generally caused by facet reflections and mode profile mismatch, is of critical importance especially for quantum information processing applications. For this purpose, we introduce linear tapering sections of LNOI waveguide. Once optimized, such a structure has potential to replace butt beam coupling with almost lossless adiabatic mode conversion [4]. We used FDTD simulation to estimate the in/out-coupling losses. Supplementary Figure S2 shows the simulation geometry. Only the TE-like modes, whose major electric field components are parallel to the optical axis of the crystal [5], were considered. In simulation, the smallest mesh size was set to be 10 nm, and the perfectly matched layers were placed far apart from the waveguide, ensuring numerical convergence and accuracy. We took into account the refractive-index changes caused by PE (see section S1) and the PE:LN layer thickness of 130 nm.


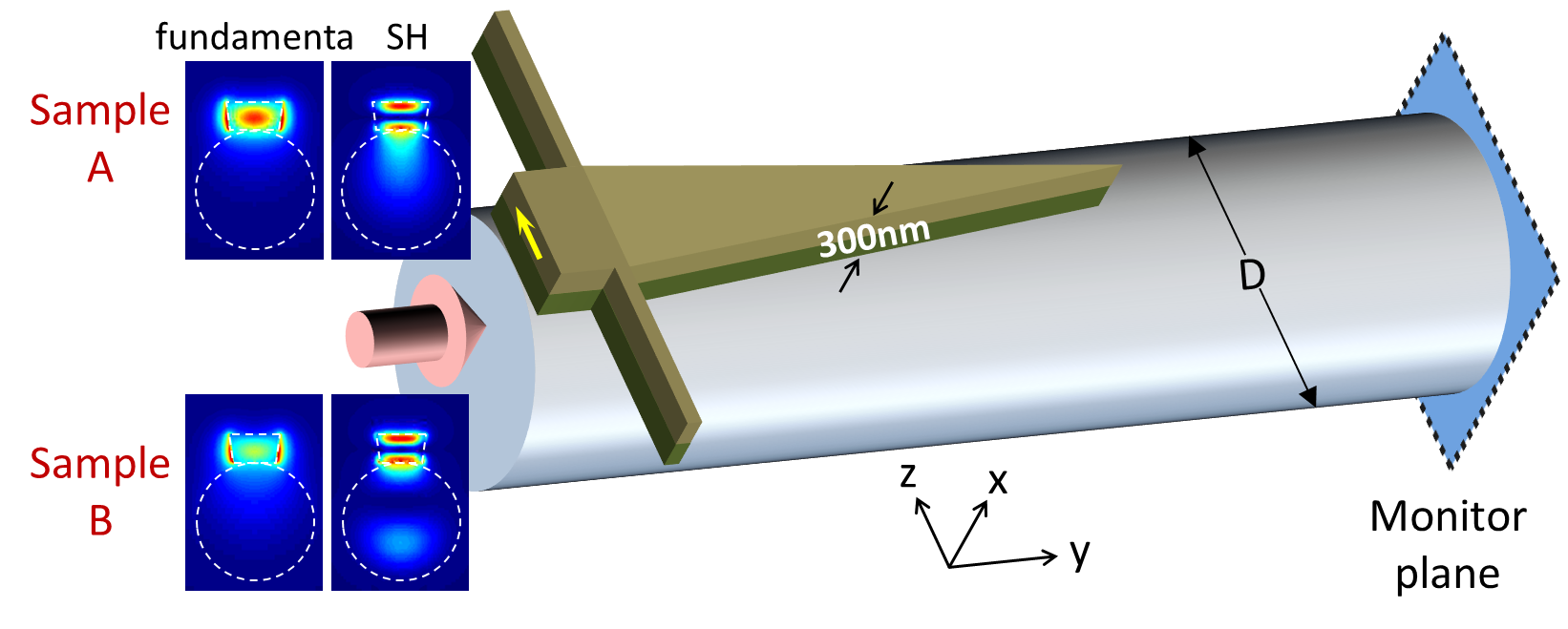


**Supplementary figure S2. FDTD simulation geometry adopted in calculation of out-coupling loss.** A TE-like guided mode is launched from the left, and the transmitted light is monitored on the right. The MF diameter D = 1.25 μm, the yellow arrow stands for the optical axis of LiNbO_3_ crystal, the green color indicates the PE:LN layer (130 nm thick), and the insets show the electric field (intensity) profiles at the fundamental and SH wavelengths (1500 nm / 750 nm) for the two samples.

For a benchmark structure having a taper length of 70 μm and no suspending arms, it is proved that the local taper length scale, $L_{t}=W\cdot(dW/dy)^{-1}$ with *W* being the local waveguide width, well exceeds the local coupling length scale, $L_{c}=2\pi/\Delta\beta$, for any pair of guided modes in the fundamental and SH harmonics. We used such a structure as reference to normalize the out-coupling losses of samples A and B.

The simulated results are shown in Supplementary Fig. S3. It is seen that our choice of taper length in experiment (12.5 μm) provides a good compromise between adiabatic mode conversion and device compactness (Supplementary Fig. S3(a)). Simulation also shows that two suspending arms bring about substantial losses especially for the fundamental harmonic (Supplementary Fig. S3(b)). The overall out-coupling loss reaches to the level of ~1 dB. We expect that as the taper length increases and the suspending arms are replaced by other structures, the out-coupling loss can decrease greatly


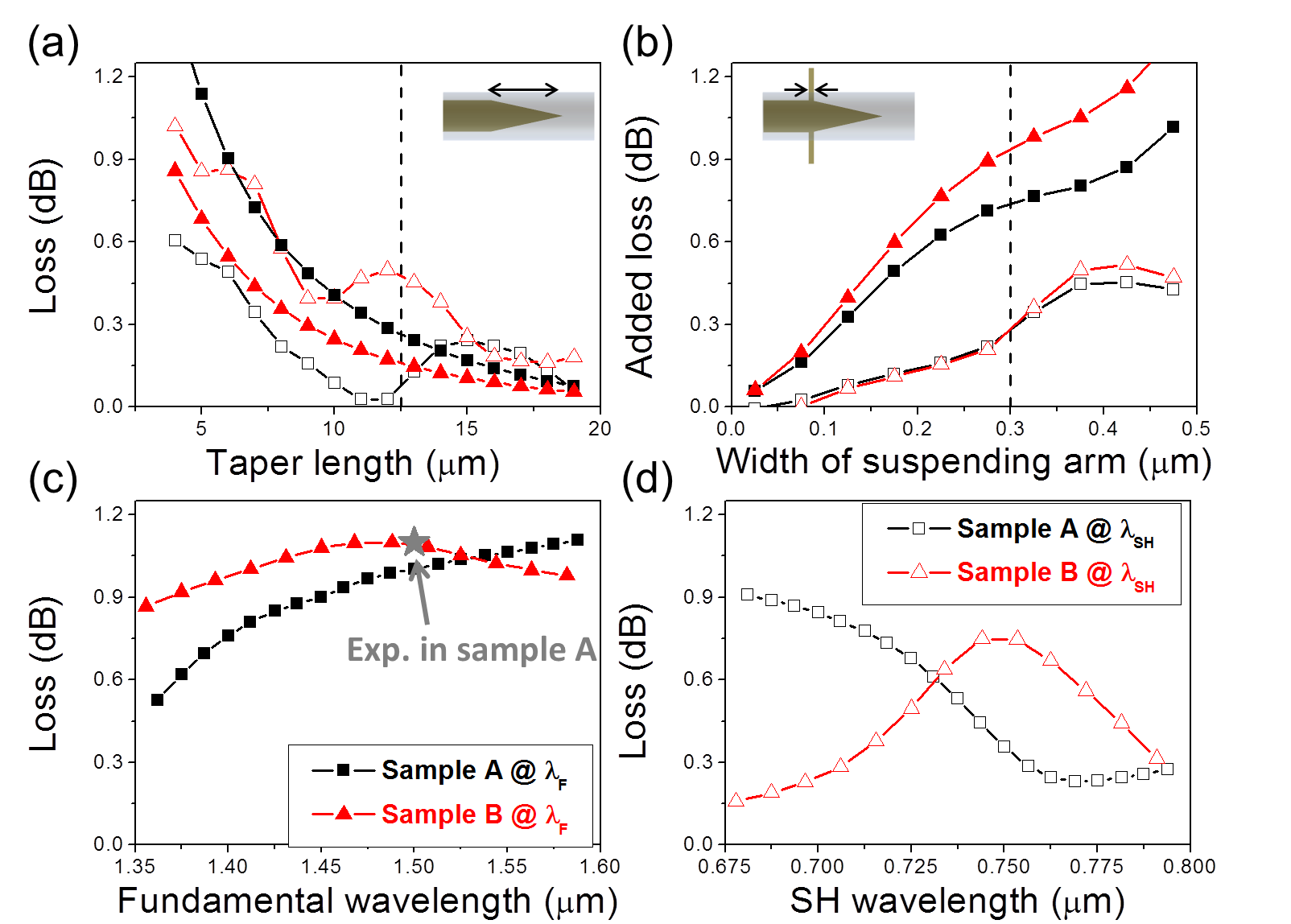


**Supplementary figure S3. Simulated out-coupling losses of samples A (black curves) and B (red curves).** The curves are functions of (a) the taper length (with no suspending arms), (b) the suspending arm width (with the taper length of 12.5 μm), and (c, d) the fundamental and SH wavelengths (with the taper length of 12.5 μm and the suspending arm width of 0.3 μm). The MF diameter is 1.25 μm. In (a, b) the wavelengths are λ_F_ = 1500 nm and λ_SH_ = 750 nm, respectively. In (c, d) the corresponding guided modes are used. The star point in (c) represents an experimental measurement for sample A.

For the fundamental harmonic, the in- and out-coupling losses are the same because both the MF-LNOI structure and the MF are single-mode. For the SH light generated in the waveguide, however, only the out-coupling loss matters. The spectra of the simulated out-coupling losses of the two harmonics are shown in Supplementary Figs. S3(c,d) and have been used to derive the normalized SHG efficiency from the raw experimental data, (see Figs. 4(b,d) in main text). We also measured the transmitted powers at the wavelength of 1500 nm before and after an MF being attached on sample A. The output powers of 8.5 mW (before attaching) and 4.9 mW (after attaching) give the in/out-coupling loss of the MF-LNOI structure ~1.1 dB (the star symbol in Supplementary Fig. S3(c)), agreeing well with the simulated value (1.0 dB)

**S3. Device re-configurability**

Our MF-LNOI structures use van der Waals and electrostatic attraction to keep components together [5]. The modest strength of the attraction makes it possible to assemble and disassemble these two parts reversibly. This post-fabrication re-configurability is valuable for nano-photonic devices because of inevitable nanofabrication inaccuracy. To demonstrate this merit of our platform, we fabricated another free-standing LNOI waveguide (200 μm long) as shown in Supplementary Fig. S4(a). Attaching one MF on it (Supplementary Fig. S4(b)), we can smoothly slip the MF along the LNOI waveguide without fall-off. By this means, we demonstrate an ultra-compact degree of freedom for device adjustment by virtue of mechanical movement [6], instead of conventional electro-optical and thermo-optical tunings.

**Supplementary figure S4. Assembly of a MF-LNOI hybrid waveguide.** Optical microscope images of a testing LNOI waveguide (a) before and (b) after attaching with an MF. The LNOI waveguide is 480 nm wide, and the MF diameter is ~1 μm.

For current proof-of-concept demonstration, we have carried out SHG experiments in a simple setup (see Fig. 4(a) in main text), consisting of two xyz stages and one optical microscope. Detaching and re-attaching MF to LNOI waveguide in the same place will not change the SHG spectrum. After alignment and adjustment, the pigtails of the MF and the substrate of the waveguide can be stuck to a common base plate for enhanced mechanical stability.

**REFERENCES:**

1. R. Ulrich and R. Torge, “Measurement of thin film parameters with a prism coupler,” *Appl. Opt.* **12**, 2901-2908 (1973).
2. M. L. Bortz and M. M. Fejer, “Annealed proton-exchanged LiNbO_3_ waveguides,” *Opt. Lett.* **16**, 1844-1846 (1991).
3. R. Ramponi, M. Marangoni, and R. Osellame, “Dispersion of the ordinary refractive-index change in a proton-exchanged LiNbO_3_ waveguide,” *Appl. Phys. Lett.* **78**, 2098-2100 (2001).
4. J. D. Love, W. M. Henry, W. J. Stewart, R. J. Black, S. Lacroix, and F. Gonthier, “Tapered single-mode fibres and devices. Part 1: Adiabaticity criteria,” *IEE Proc.-J: Optoelectron* **138**, 343-354 (1991).
5. A. V. Gorbach and W. Ding, “Microfiber-lithium niobate on insulator hybrid waveguides for efficient and reconfigurable second-order optical nonlinearity on a chip,” Photonics 2, 946-956 (2015).
6. M-K. Kim, I-K. Hwang, M-K Seo, and Y-H. Lee, “Reconfigurable microfiber-coupled photonic crystal resonator,” *Opt. Express* **15**, 17241-17247 (2007).
